# A synergistic approach to tooth remineralization using nano-chitosan, fluoride, and pulsed magnetic field

**DOI:** 10.1038/s41598-025-99607-3

**Published:** 2025-05-08

**Authors:** Alaa M. Khalil, Samar A. Abbassy, Mona Mohy ElDin, Sherif Kandil, Ahmed M. El-Khatib

**Affiliations:** 1 Basic Sciences Department, Faculty of Engineering, Pharos University in Alexandria, Alexandria, Egypt; 2 Alexandria Dental Research Center, Alexandria, Egypt; 3 Dental Biomaterials Department, Faculty of Dentistry, Alexandria University, Alexandria, Egypt; 4 Materials Science Department, Institute of Graduate Studies and Research, Alexandria University, Alexandria, Egypt; 5 Physics Department, Faculty of Science, Alexandria University, Alexandria, Egypt

**Keywords:** Enamel remineralization, Magnetic field, Chitosan nanoparticles, Fluoride, Regenerative dentistry, Biophysics, Nanoscience and technology

## Abstract

Globally, dental caries remains a health concern due to their complications of pain, infection, and tooth loss. The traditional dental remineralization by using fluoride is effective but limited in advanced caries and continued treatments. While Calcium phosphate is beneficial in restoring mineral, it needs external aids to be effective. This research explores a synergistic approach to enhance tooth remineralization for a total of 72 samples by harnessing the effects of nano-chitosan, fluoride, and exposure to magnetic fields. The nano-chitosan solution is prepared using an ionic interaction method, initially without fluoride, and is subsequently mixed with fluoride at concentrations of 0.05% and 2%. The structural and morphological properties of the prepared nano-chitosan were confirmed using SEM, XRD, and FTIR. The samples were exposed to a pulsed magnetic field (PMF) of 18 mT ± 2% to assess its effect on remineralization. Demineralized teeth samples are treated by synthesized agents combined and free of exposure. Treatment efficacy is evaluated using XRD, EDX, SEM, and the Vickers microhardness test. The results showed optimal enhancement of dental enamel treated by 0.05% fluoride and ChNPs with the aid of exposure. Its morphology showed new mineral layers, likely fluorapatite, and it had the highest Ca/P ratio and maximum VHN value. These findings support the possibility of prevention of early developed lesions of teeth by this non-invasive technique with low cost. Commercially, it is suggested to assess the benefit of using PMF combined with remineralizing agents instead of high-cost materials in domestic settings.

## Introduction

The human tooth is composed of highly mineralized tissues of the body, containing hydroxyapatite as the primary constituent. Dental hard tissues are continuously undergoing cycles of demineralization and remineralization. A drop in the pH of the oral cavity results in demineralization, which, if continued, leads to loss of minerals from tooth structure, resulting in dental caries. The reversal can occur if pH rises, resulting in the deposition of calcium, phosphate, and fluoride^1,2^. One of the most significant health concerns worldwide is dental caries, which is commonly known as tooth decay. The natural repair mechanism of enamel for caries prevention and restoration of tooth structure has arisen great concern recently^3,4^. Dental caries is a dynamic process that occurs when demineralization exceeds remineralization. The progression of dental caries is a slow process, and non-invasive intervention during early stages can convert the lesion to an inactive state. Early diagnosis of incipient lesions can lead to a new era in preventive dentistry as remineralization. The best mode for caries management is the use of remineralizing products^5^. The naturally occurring mineral form of calcium apatite is hydroxyapatite (Ca_5_(PO_4_)_3_(OH)). It is very important for building tooth enamel and the remineralization of demineralized enamel areas.

Fluoride is considered as most effective agent in the prevention of dental caries; it helps to enhance remineralization of that lesions^5^. The supplement of fluoride as varnishes, gels, fluoride-releasing restorative materials, and oral healthcare products exerts an anti-caries effect. It has resulted in significant improvements in the incidence of dental caries. Fluoride reduces the solubility of enamel through the substitution of hydroxyl ions to form fluorapatite or the partial substitution to form fluorohydroxyapatite. Both forms have a lower solubility than hydroxyapatite^6^. Chitosan is a copolymer of glucosamine and N-acetyl glucosamine produced from the partial deacetylation of chitin. Its antimicrobial properties, biocompatibility, ability to promote tissue regeneration emerged it as a promising material for dental applications^7^. The higher surface-to-volume ratio and enhanced properties of nano chitosan gave a chance to facilitate the deposition of calcium and phosphate ions into the tooth surface. Therefore, it enhances remineralization and the mechanical properties of demineralized enamel^8^. The urge to use electromagnetic fields has been a subject of interest in dentistry and oral care as a new medication technology^9^. Yet, it is not fully utilized, and its mechanism of interaction is not fully understood^10^. Although studies have shown that electric and magnetic fields enhance penetration of ions for local delivery to enamel and dentin, and may modulate cellular activities, including mineralization processes^11,12^. The combination of Nano chitosan, fluoride gel, and exposure to magnetic fields synergistically may exhibit a novel opportunity to enhance tooth remineralization. The concurrent use of modulating cellular activities because of exposure to magnetic fields, the antibacterial potential of nano chitosan, and the promotion of enamel with fluoride ions results in an increase in the efficacy of these interventions. As a result, it holds promise for enhancing tooth remineralization and combating dental caries. This study investigates the synergistic effect of nano chitosan, fluoride gel, and magnetic fields on tooth remineralization.

## Materials and methods

### Materials

In the experiment, Chitosan, Product number 448,877, medium molar mass Chitosan (180–200 kDa) with a degree of deacetylation of 75–85% and Sodium tripolyphosphate (TPP) (85%) Product number 238,503 are purchased from Sigma alderich. Acetic acid (assay of ≥ 99.8%) is received from Merck, USA. Sodium Fluoride (NaF) (99.99%) Product number 91.06450.0025 is received from Merck, Germany. EDTA (19%) is received from Meta Biomed Korea. Sodium Fluorine (NaF) (assay of ≥ 97%) are purchased from Vetec. Deionized water without further purification.

### Preparation nano-chitosan

The Nano-chitosan (ChNPs) solution is prepared by using the ionic interaction method as adapted by *Ebrahimi N.et al.*^13^. Chitosan powder 5 mg is dissolved in 250 ml of 1% acetic acid aqueous solutions under magnetic stirring at 500 rpm/min at room temperature for 20–24 h, until a clear solution is obtained. The solution is raised to pH 4.6–4.8 with NaOH^13–15^. TPP solution of 0.1% is prepared by dissolving 0.01 g of TPP in 10 ml of deionized water. TPP solution is added dropwise with a syringe to the formerly prepared chitosan solution under magnetic stirring at 800 rpm and continued for 30 min stirring at room temperature to form ChNPs solution. The ChNPs solution is adopted by the ionic interaction method as the positively charged amino groups is interacted with the negatively charged TPP.

### Preparation of nano-chitosan-fluoride composite

Further, the Nano-Chitosan-Fluoride (ChNPs-F) composite is prepared by dissolving a solution of Sodium Fluoride (NaF) of concentration 12 mg/ml in distilled water and 19 mg of TPP dissolved in 5 ml distilled water is mixed and diluted by a 1/5 factor. The NaF solution is added in two concentrations (0.05% and 2% m/v) with 900 ppm of fluoride to the prepared ChNPs solution (5 mg/mL) and kept under mechanical stirring for 30 min^16^. After homogenization, the TPP solution is dropped, and the final mixture is placed under high rotation for 10 min at 3500 rpm (LMC-4200R). The ChNPs-F solutions were stabilized at room temperature (~ 20 °C) overnight prior to further measurements^13^.

### Nano-conformational analysis

The Nano-confirmation of prepared ChNPs and solutions of ChNPs-F composite are maintained by analyzing scanning electron microscopy (SEM) (Jeol IT 20), X-ray diffraction spectroscopy (XRD) and Fourier transforms infrared spectroscopy (FTIR) (THERMO NICOLET, USA)^15^.

### Exposure to pulsed magnetic field methodology

**Fig. 1:**
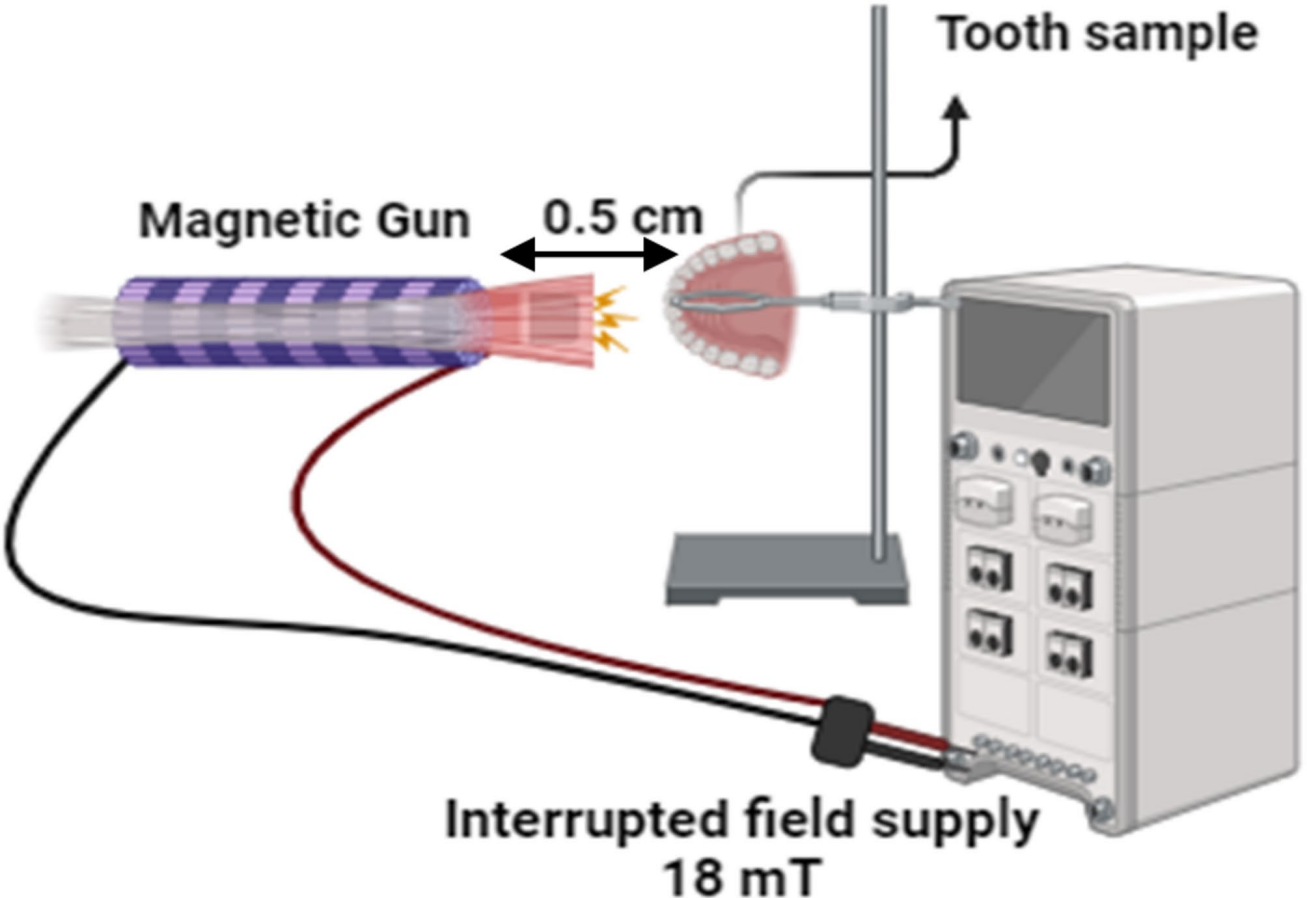
The schematic diagram of tooth sample PMF exposure system.

The exposure facility is performed by a pulsed magnetic field (PMF) system to apply an interrupted field by using a magnetic gun (a device that uses PMF for precise control and direct field localization) of 1020 turns with a total resistance of 11.2 W and a core length of 8.5 cm. The magnetic coil was connected to a 65 mA power supply via an electronic switching device, operating at a 50% duty cycle. The tooth samples under investigation are placed 0.5 cm away from the end of the solenoid coil as shown in Fig. 1. The PMF intensity at the point of exposure (at the midpoint of the specimen) is measured by using a model 4048 Gauss/Tesla meter, with a T-4048.001 probe (USA) with an accuracy of ± 2%, and is found to be 18 mT. The current field intensity indeed builds upon the foundation laid by other previous studies conducted in our lab^11^. The chosen field intensity value was within the safe limits that have been stated by the International Commission on Non-Ionizing Radiation Protection^17^.

### Treatment protocol

**Fig. 2:**
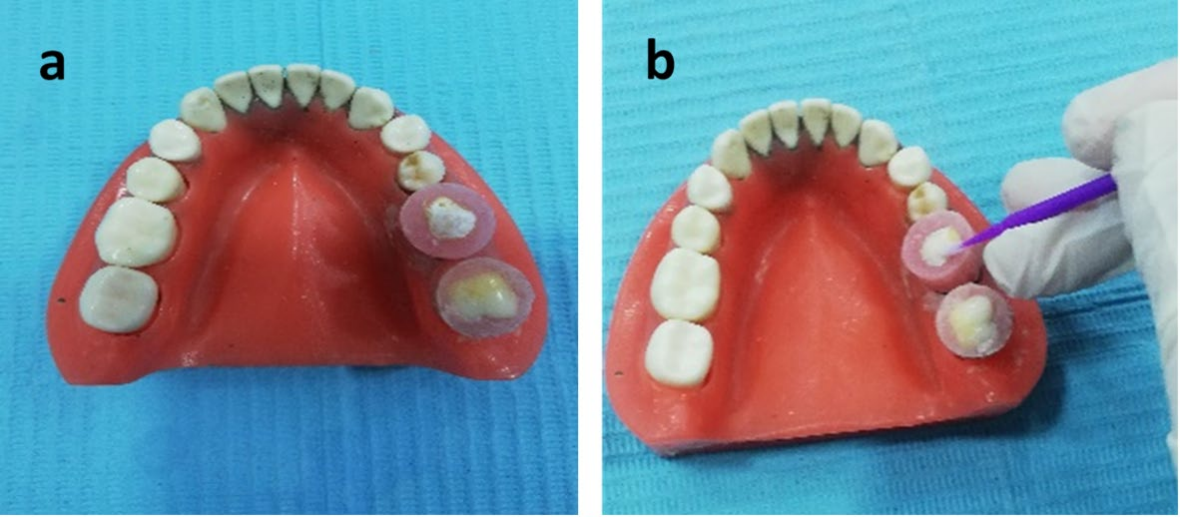
A) Teeth samples showing its demineralized labial surface, b) teeth samples showing its varnished labial surface.

**Fig. 3:**
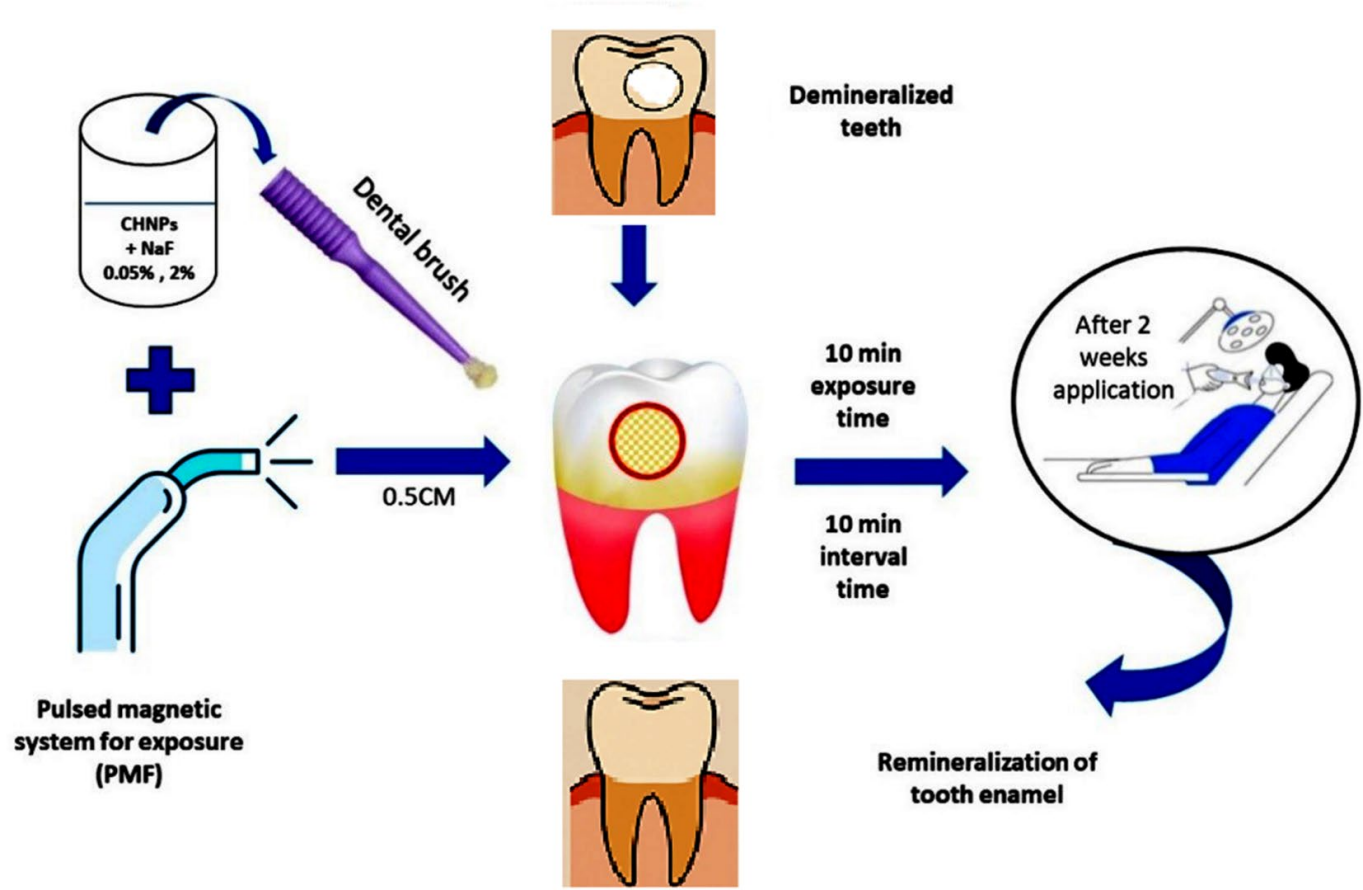
Schematic diagram for the treatment protocol.

The sample size for each treatment group is 10 samples, for a total of 72 samples to ensuring robust statistical analysis and comprehensive characterization of remineralization effects^18^. For teeth sample preparation, extracted human permanent premolars are obtained from patients requiring extraction as a part of their dental treatment at the faculty of Dentistry, Alexandria University. No formal ethical review is needed in the study because it involved the use of de-identified tissue obtained during routine clinical procedures, and no personal identifying information is disclosed. After teeth extraction, samples were inspected free from any lesions, cracks, or white spots visually by using bright light and a magnifying mirror to be selected for the further treatments. The premolars are collected and maintained in an artificial saliva solution. The ionic composition of the artificial saliva solution is methyl-p-hydroxybenzoate (2.00 g), sodium carboxymethyl cellulose (10.00 g), potassium chloride (KCl) (0.625 g), magnesium chloride hexahydrate (MgCl₂·6 H₂O) (0.059 g), calcium chloride dihydrate (CaCl₂·2 H₂O) (0.166 g), dipotassium phosphate (K₂HPO₄) (0.804 g), monopotassium phosphate (KH₂PO₄) (0.326 g) and Distilled Water up to 1 L^18^. Samples are disinfected by 3% sodium hypochlorite to remove adhered bacteria prior to cutting, and then teeth are embedded in acrylic resin blocks. On the purpose of simulating demineralization and enamel white spot lesion, a labial surface of each specimen is treated by the demineralizing solution (calcium chloride (CaCl_2_. 2H_2_O) 2.2 mmol/l, potassium dihydrogen phosphate (KH_2_PO_4_. 7H_2_O) 2.2 mmol/l, and lactic acid 0.05 mmol/l) at pH 4.5 with 50% sodium hydroxide (NaOH)^18^. Teeth samples are immersed in demineralizing solution (one sample/30 mL) for 7 days and then incubated in artificial saliva at 37 °C for the 2 weeks’ period of the study^19^. The chosen demineralization protocol is designed to generate controlled, early-stage enamel lesions while preserving structural integrity for downstream analyses as adopted elsewhere^19^. This approach in alignment with established models using lactic acid^20^ and Silver Diamine Fluoride^21^ and accounts for enamel’s slower demineralization kinetics compared to dentin. Furthermore, teeth samples are stored in distilled water until used, whereas the solution is changed every 24 h. The crowns of all teeth are separated from the roots by a diamond-coated bandsaw under continuous water cooling, then stored in artificial saliva at pH 7. A specially fabricated circle plastic mold with an internal diameter of 10 mm and 20 mm in height is fabricated. A separating medium is used to coat the internal surface of the mold. The mold is filled with self-curing acrylic resin; the base of the mold rested on a glass slab in order to obtain a flat, smooth surface base. Each crown is embedded horizontally in the middle of the mold containing self-cure acrylic resin in the dough stage while the lingual surface is embedded in resin and leaves about two mm from the labial surface projecting above the surface of the mold using a caliper as shown in Fig. 2a. Then the labial surface of all the teeth is painted with nail varnish except for a two mm square in the middle of the crown (mesio distally and cervico incisally) as shown in Fig. 2b. The treatment protocol is maintained as follow: the remineralization treatments are carried out by brushing the labial surfaces of specimens with a soft toothbrush with solutions of ChNPs, NaF 0.05% and NaF 2% each alone and mixed with them as presented in the schematic diagram shown in Fig. 3. The brushing is maintained with minimum pressure and carried out three times daily for two weeks. The brushing was applied on a confined area of the tooth surface with minimal pressure simulating that applied during tooth bonding by dental bonding brush (Brush & Bond Standard Activator Microbrushes). The teeth samples under investigation are placed in a jaw-like model that is placed 0.5 cm away from the end of the solenoid coil, as formerly shown in Fig. 1. The exposure to PMF is maintained for 10 min at frequency 1.5±0.02 Hz three times for 10 min intervals. The chosen frequency is adopted as reported elsewhere by Moiseeva NS et al.^22^.

### Teeth characterizations

The treated teeth specimens from each group are analyzed elementally by energy-dispersive X-ray spectroscopy (EDX) and examined by SEM, and XRD^23,24^. The spectra of EDX were collected at specific energy ranges for elemental analysis of Ca: 3.0–5.0 keV, P: 2.0–3.0 keV and F: 0.5–1.0 keV. X-ray diffractometer type Bruker D2 Phaser of Cu Kα radiation source was used at wavelength of 1.54 Å and 2θ range 5° to 90°. The microhardness of the teeth specimens is obtained by using a Vickers microhardness tester (402 MVD, Wolpert-Wilson Instruments) and introduced as Vickers hardness numbers (VHNs)^18^. The VHNs test was maintained superficially at 0.5 kg load, 10 s dwell time and 3 times indentations. On the basis of the characterization test, the teeth samples for each group are divided into two sets. One set of 5 samples is used for XRD analysis without damaging it and followed by EDX and SEM tests and the other set of 5 samples is used for hardness testing due to its destructive nature.

### Statistical analysis

Statistically, all quantitative data of tooth samples were described using mean and standard deviation for normally distributed data. The SPSS for Windows statistical package program (SPSS Inc., version 21) was used. The obtained results are presented as mean ± standard deviation. Each test was performed at least three times with a minimum of three samples per termination point. The independent *t-test* was used for comparison between two independent populations, while comparisons involving more than two populations were analyzed using ANOVA (Analysis of Variance). The statistical significance was defined by a post hoc Tukey’s test, and *p* < 0.05 was considered significant. This study is aimed to highlight statistically significant patterns within the data, rather than to quantify the magnitude of differences between groups.

## Results

The synthesized ChNPs solutions by using an ionic interaction method, initially without fluoride, and subsequently combined with F 0.05 and F 2% are characterized. Characteristically, the structural confirmation is maintained by analyzing SEM, XRD and FTIR. On the other hand, the demineralized teeth samples are brushed by a soft toothbrush with solutions of ChNPs, NaF 0.05% and NaF 2% each alone and mixed of them free of exposure to PMF and combined with PMF. Then after, the treated teeth specimens from each group are analysed morphologically by XRD and SEM, elementally by EDX, and mechanically by VHN.

### SEM micrographs for synthesized ChNPs solutions

**Fig. 4:**
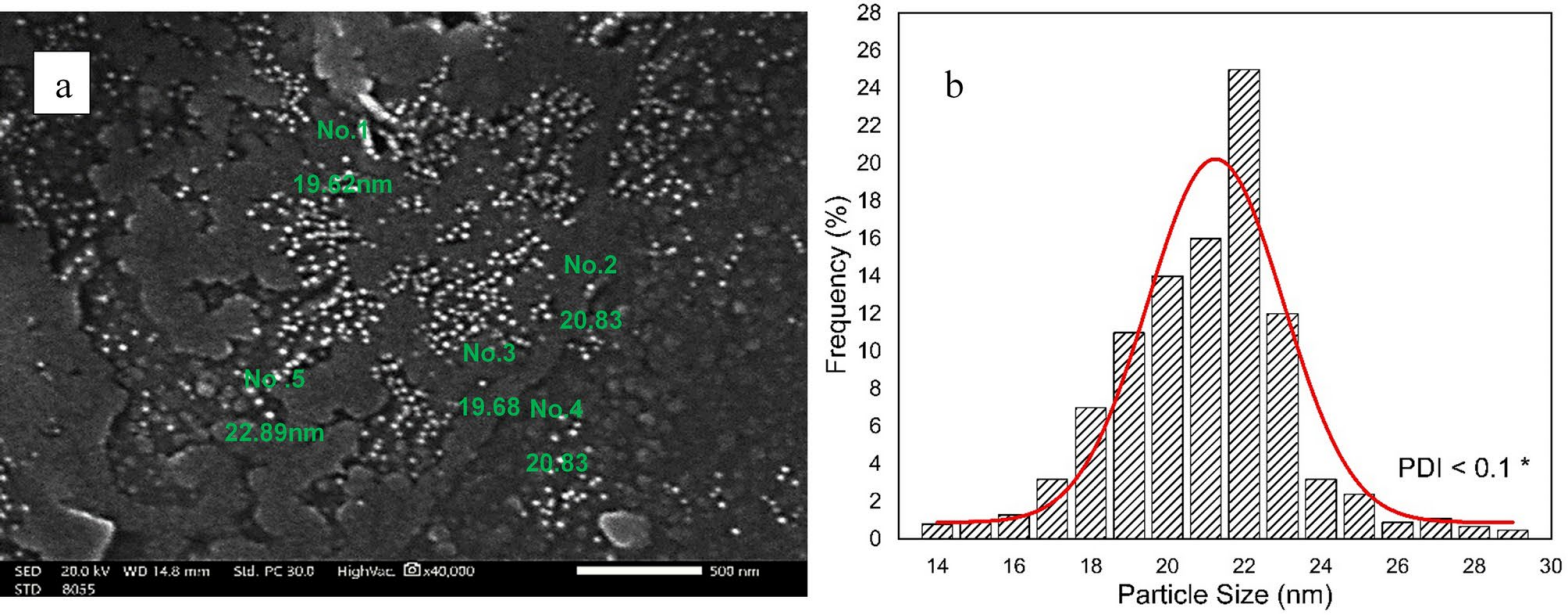
(a) SEM (2000X) graph of ChNPs with average diameter size of 21.253 ± 0.194 nm, (b) histogram of ChNPs diameter sizes distribution (PDI < 0.1).

**Fig. 5:**
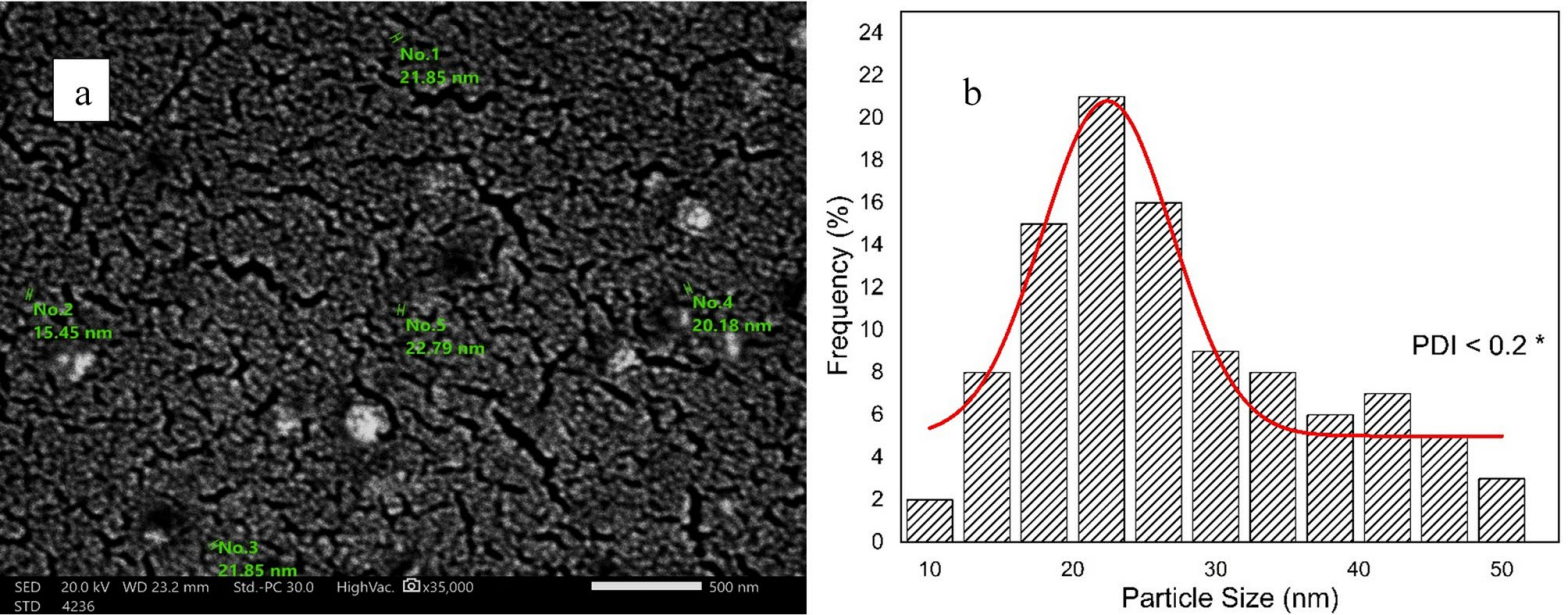
(a) SEM (2000X) graph of ChNPs-F with average diameter size of 22.416 ± 0.558 nm, (b) histogram of ChNPs-F diameter sizes distribution (PDI < 0.2).

The photographs of synthesized ChNPs are presented in Fig. 4 and they confirm the formation of well-distributed organized particles of circular shape as illuminated in Fig. 4a. It revealed the average diameter size of ChNPs to be 21.253 ± 0.194 nm with PDI < 0.1, as presented in the histogram diameter size distribution shown in Fig. 4b. Moreover, the ChNPs diameter sizes in combination with NaF are confirmed, as shown in Fig. 5a. It shows the interspersing of fluoride atoms over the scanned section and the presence of circular-shape ChNPs with an average diameter size of 22.416 ± 0.558 nm and PDI < 0.2 as presented in Fig. 5b.

**Fig. 6:**
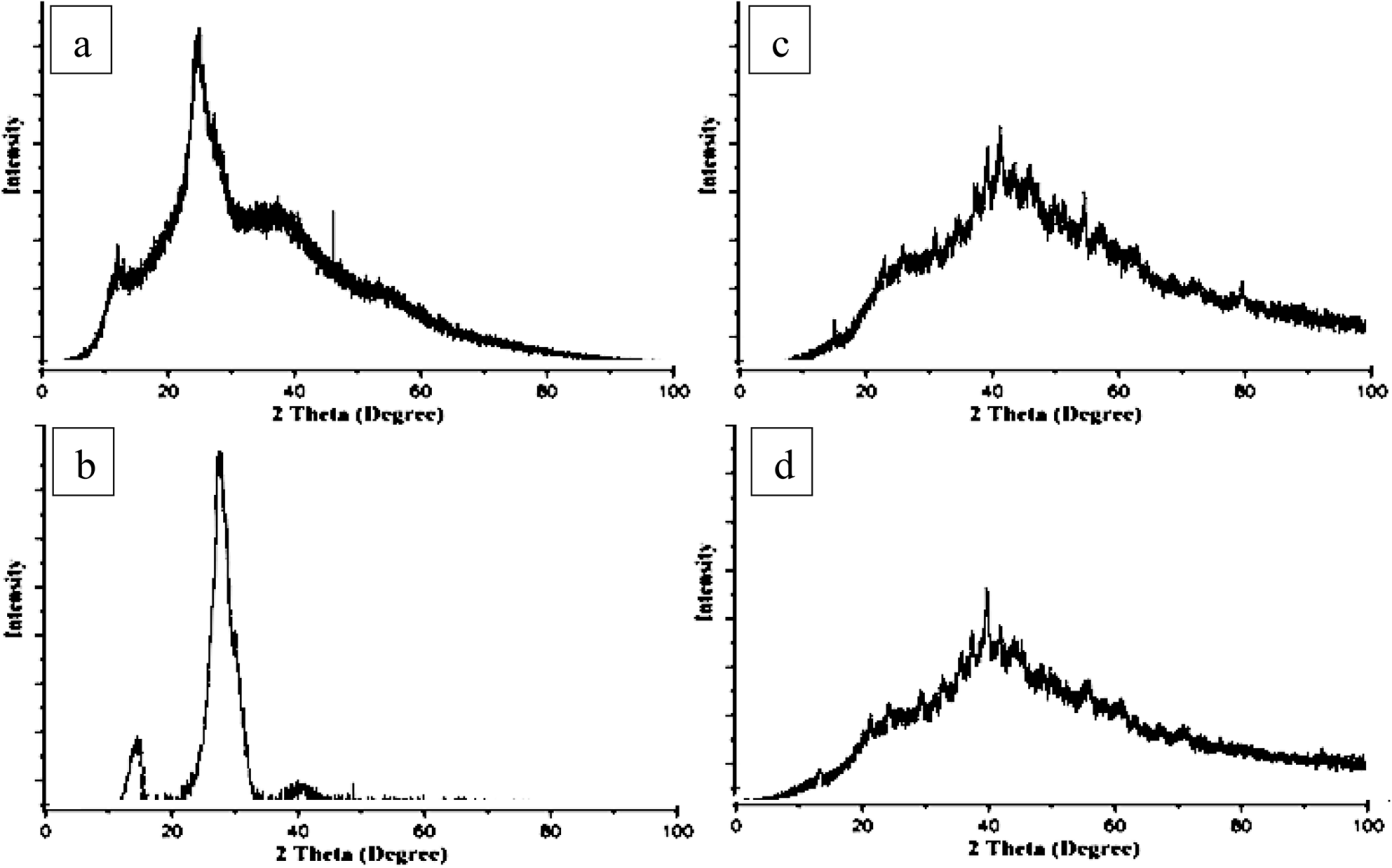
XRD diffractograms of (a) ChNPs solution free of NaF, (b) NaF free of ChNPs, (c) ChNPs solution mixed with 0.05%NaF and (d) ChNPs solution mixed with 2%NaF.

In addition, the structural confirmation is obtained by XRD diffractograms shown in Fig. 6 for ChNPs solution free of NaF, NaF free of ChNPs and mixed ChNPs with 0.05% and 2% NaF as presented in Fig. 6a–d, respectively. The XRD pattern of ChNPs exhibited two characteristic broad diffraction peaks at 2θ around 9.63 and 20.53 that are typical fingerprints of semi-crystalline chitosan as indicated in Fig. 6a. The peaks around 9.63 and 20.53 are related to the crystals in the chitosan structure and both of these peaks attribute a high degree of crystallinity to the prepared ChNPs^25^. The lower intensity exhibited by the diffraction peaks revealed that they are amorphous in nature. The ionic interaction between TPP and NH3 + of chitosan molecules has resulted in the formation of ChNPs. The intensity of diffraction peaks was increased as a consequence of transforming amorphous chitosan into crystallized form after reaction with TPP^26^. The X-ray diffraction pattern for sodium fluoride shown in Fig. 6b reflected prominent sharp peaks that match with the expected NaF standard XRD peaks corresponding to specific 2θ angles, confirming its crystalline nature. No other crystalline phases detectable by XRD were found indicating the purity of NaF^27^. Regarding XRD analysis of ChNPs with varying concentrations of sodium fluoride (NaF) at 2% and 0.05% shown in Fig. 6c, and d illuminated no distinct differences in the crystallinity, particle diameter size, and structural properties of the resulting composites. Specifically, the incorporation of NaF into ChNPs tends to exhibit broader and less intense peaks in XRD, suggesting reduced crystallinity and possible amorphous characteristics^28^.

### FTIR spectra for synthesized ChNPs solutions

**Fig. 7:**
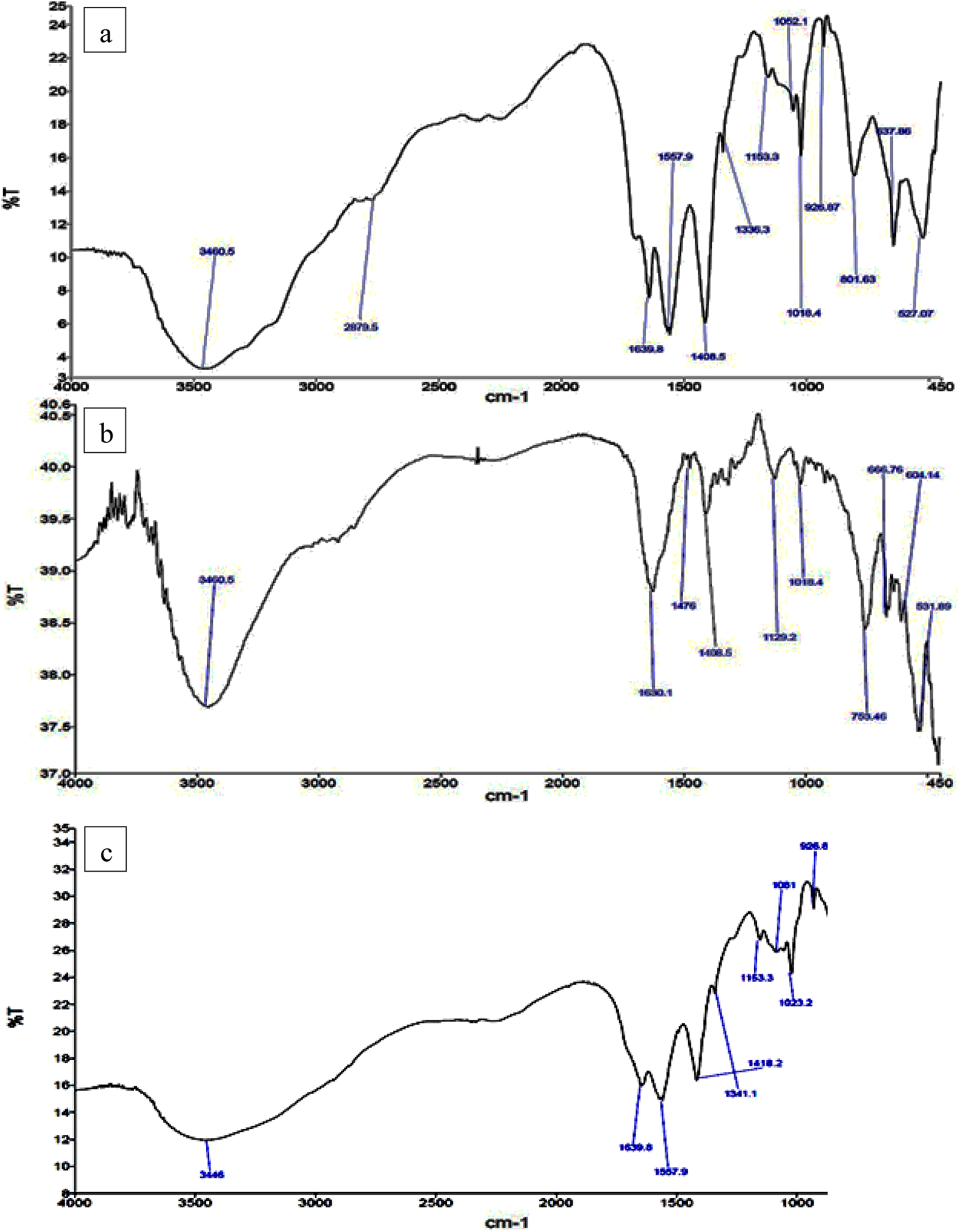

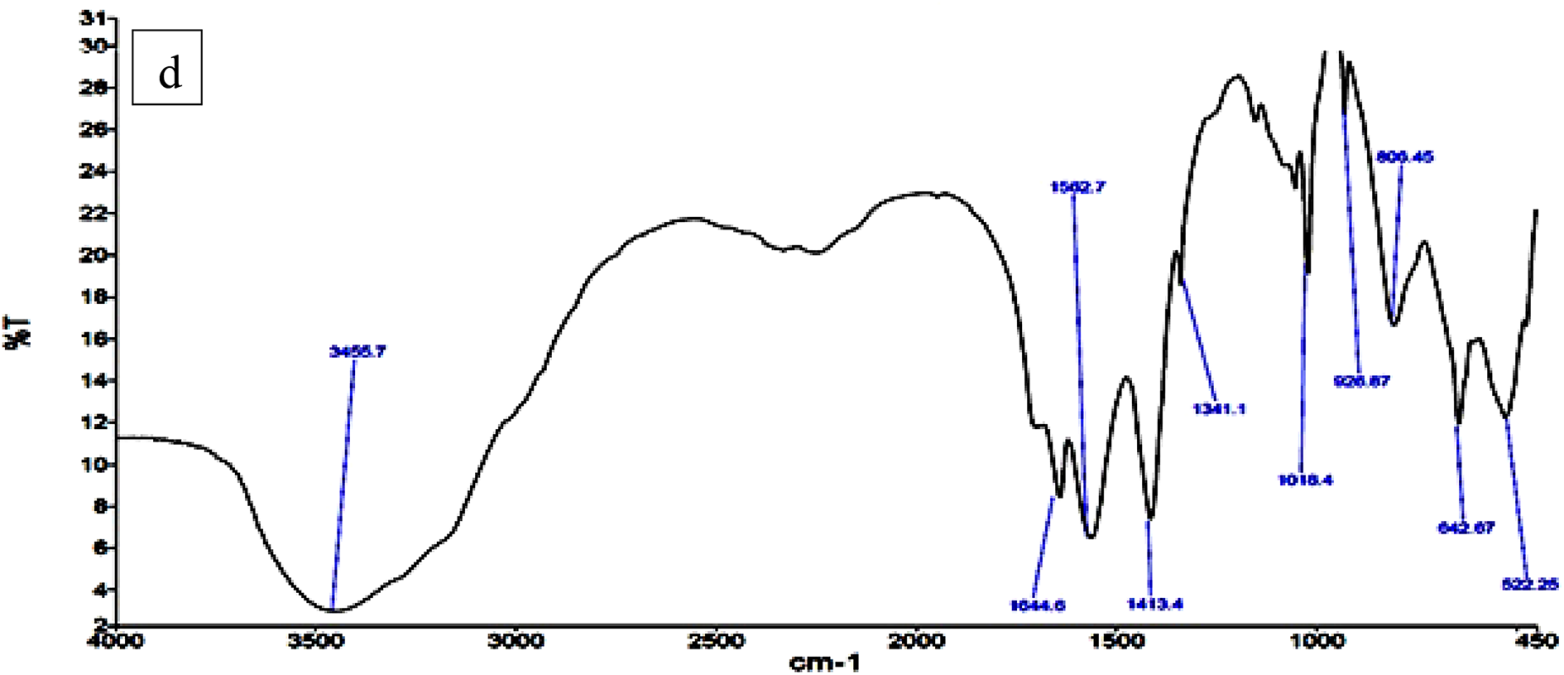
Set of FTIR spectrum for (a) ChNPs solution free of NaF, (b) NaF free of ChNPs, (c) ChNPs solution mixed with 0.05%NaF and (d) ChNPs solution mixed with 2%NaF.

The former synthesized solutions are confirmed by FTIR spectra as shown in Fig. 7. The FTIR spectrum of the ChNPs solution, Fig. 7a, showed a stretching vibrational peak at ~ 3600 cm^–1^ confirms the presence of a free hydroxyl group (OH). At ~ 3400 cm^–1^ a vibrational peak appeared, indicating that an amino group is present. Another broad band observed at ~ 3300 cm^–1^ that confirms the presence of bonded hydroxyl group, bands at 2925 cm^–1^ and 2365 cm^–1^ are given credit for the -CH_2_ groups. At 1650.80 and 1600.77 cm^–1^ it is due to the CONH_2_ and NH_2_ groups, respectively. Another important band for CH was observed at 1423.43 cm-1 owing to –CH2 wagging. FTIR spectroscopy also was employed to analyze the vibrational characteristics of the NaF as shown in Fig. 7b at whitch it indicated the bsorbance spectrum to be within the range from 4000 to 450 cm^–1^. Concerning the spectra of ChNPs with F 0.05 and F 2% solutions, band values between 3700 cm^–1^ and 3200 cm^–1^, are associated with O-H and N-H stretching vibrations as indicated in Fig. 7c and d, respectively. The band values between 3000 cm^–1^ and 3200 cm^–1^ are attributed to the overlapping of stretching of OH and NH_2_ groups, which belong to ChNPs.

### XRD diffractograms for teeth samples

**Fig. 8:**
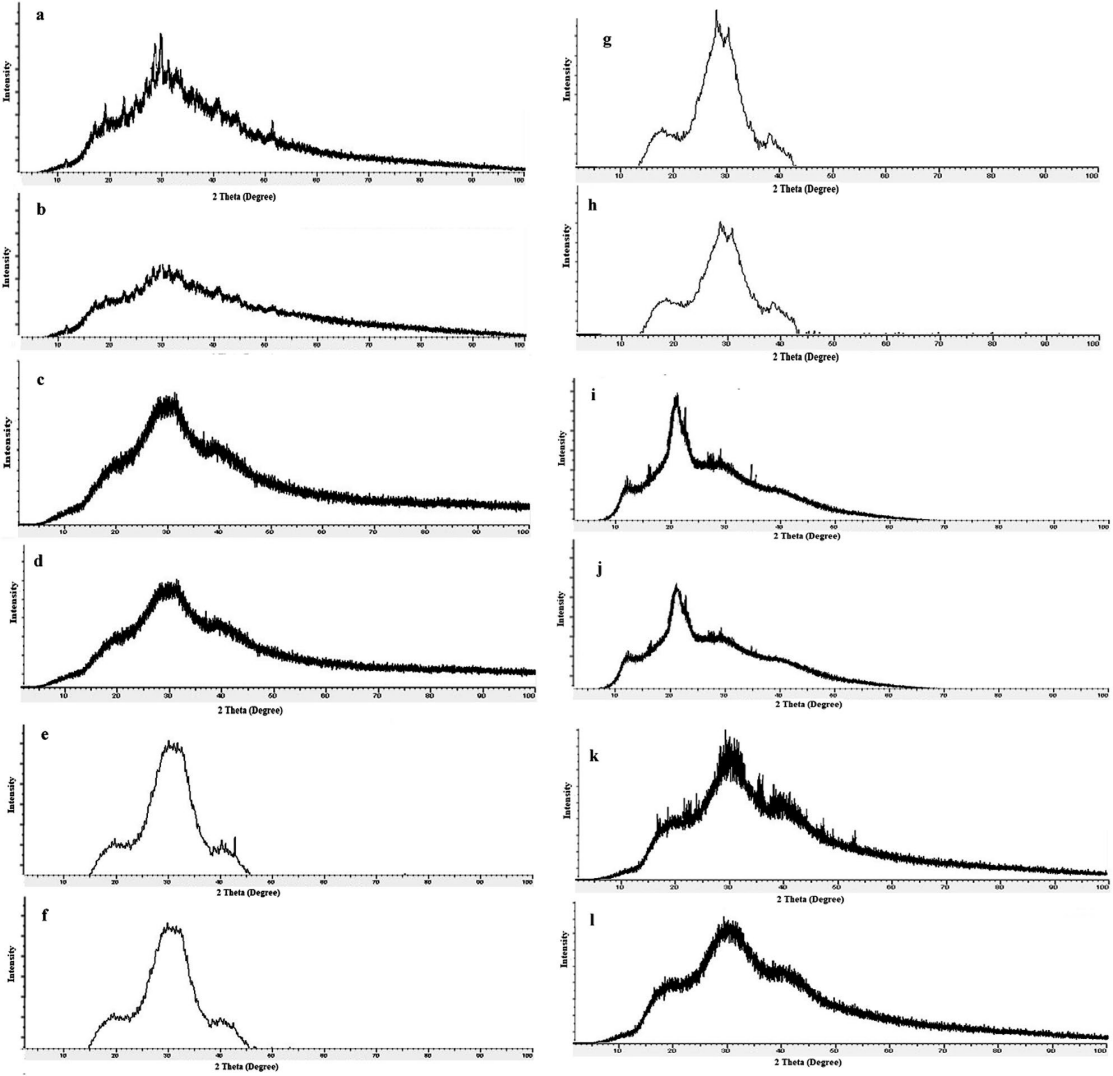
Set of XRD patterns for (a) sound teeth, (b) demineralized teeth, and teeth treated by (c) ChNPs solution only, (d) ChNPs solution and exposed to PMF, (e) 0.05%NaF only, (f) 2%NaF only, (g) 0.05%NaF and exposed to PMF, (h) 2%NaF and exposed to PMF, (i) ChNPs solution mixed with 0.05%NaF, (j) ChNPs solution mixed with 2%NaF, k) ChNPs solution mixed with 0.05%NaF and exposed to PMF, l) ChNPs solution mixed with 2%NaF and exposed to PMF.

The XRD pattern of the sound teeth samples showed a sharp and intense XRD diffraction peak that appeared in the region of 20° to 40°, with a high-intensity count as depicted in Fig. 8a. In contrast, the patterns of demineralized teeth showed broader and nonspecific peak locations as shown in Fig. 8b. On observing the diffractograms shown in Fig. 8, the treatment of enamel teeth samples with ChNPs only showed insignificant adjustments to sound with a less broad peak compared to demineralized ones, as shown in Fig. 8c. A slight improvement in enamel crystallography is obtained as a result of the combination of ChNPs supplement and exposure to PMF, as shown in Fig. 8d. The XRD pattern shown in Fig. 8d depicts a slight shift to 30° to come closer to the sound peak location and sharper than the demineralized and unexposed ones. Statistically, there are no crystallographic significant changes for teeth samples treated with 0.05% and 2% NaF, as stated in Fig. 8e and f. Step forward, the enamel samples treated by 0.05% and 2% NaF supplement and exposed to PMF showed subtle modifications to become sharper and allocated closer to the sound than demineralized teeth samples as illuminated in Fig. 8g and h. The maximal enhancements of enamel lattice structures are obtained for teeth samples treated with remineralizing agents ChNPs and NaF 0.05% alone and with exposure PMF, followed by ChNPs and NaF 0.05% alone and with exposure PMF as depicted in Fig. 8i to L.

### SEM micrographs for teeth samples

**Fig. 9:**
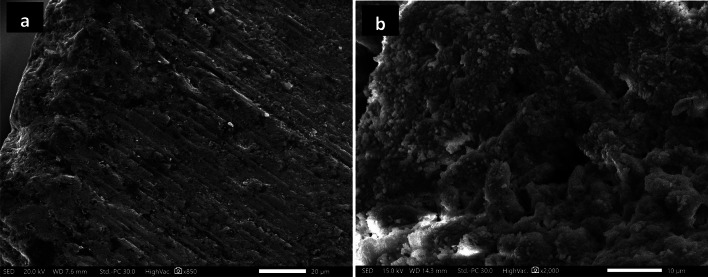
SEM images (2000×) of (a) sound enamel tooth structure and (b) demineralized tooth structure (EDTA).

**Fig. 10:**
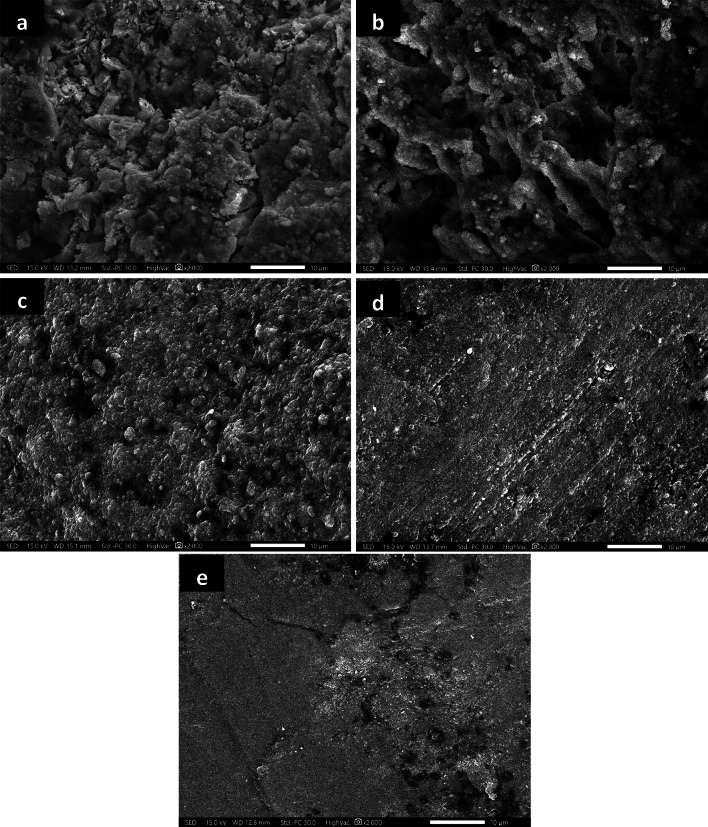
SEM images (2000×) of enamel tooth structure treated with (a) ChNPs, (b) NaF-0.05%, (c) NaF-2%, (d) ChNPs- NaF-0.05% and (e) ChNPs- NaF-2%.

**Fig. 11:**
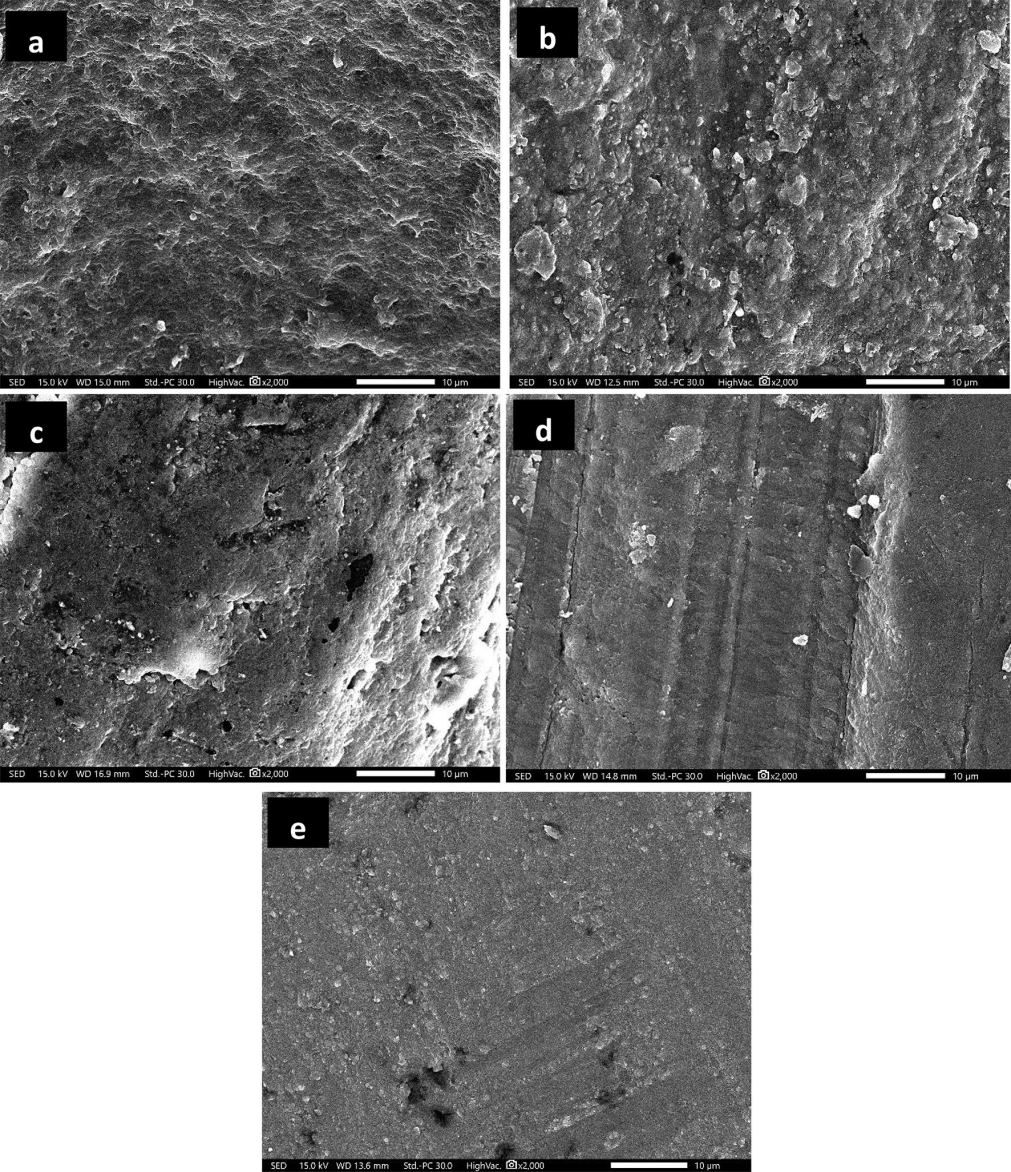
SEM images (2000×) of enamel tooth structure exposed to PMF and treated with (a) ChNPs, (b) NaF-0.05%, (c) NaF-2%, (d) ChNPs- NaF-0.05% and (e) ChNPs- NaF-2%.

The morphological analyses of all teeth samples are obtained from studying the SEM photographs shown in Figs. 9, 10 and 11. The SEM photograph of sound enamel shown in Fig. 9a illuminates dense lines as enamel rods that run from dentine in a slightly curved pattern to the enamel surface. Internally, there are lower calcified dense inter-rods of less density placed between enamel rods. On the contrary, the obtained SEM photograph of demineralized enamel shown in Fig. 9b indicates remarkable surface changes evidenced by the fragmentation and discontinuities of enamel crystals to be like an unfinished puzzle. The teeth samples treated by ChNPs and fluoride are carefully photographed, and SEM images are presented in Fig. 10a–e. The obtained SEM images of all teeth samples treated by ChNPs, ChNPs-F 0.05% and ChNPs-F 2% relative to the demineralized teeth samples showed remarkable enhancement in its structural cementation as noticed in the reduction of surface irregularities and an increase in mineral content. The Fig. 10a, d and e illuminated the ability of ChNPs to act as scaffolds aiding in the controlled release and deposition of minerals. The impact of fluoride supplements is clearly observed on the demineralized teeth surfaces covered by nanocrystals that aggregated into micro-clusters arranged in a thick apatite layer, as shown in Fig. 10b and c. Significant synergism of supplement of fluoride is obtained clearly in the demineralized teeth samples as it become smoother, more uniform and it has homogenous enamel surfaces as shown in Fig. 10d and e. On the other hand, the exposure to PMF influences the teeth structures including dental demineralization as clearly observed in Fig. 11a–e. Significantly, a reduction in tooth surface roughness, fewer micro-cracks, and restoration of enamel surface are obtained for teeth samples supplemented with ChNPs and exposed to PMF which as shown in Fig. 11a. Improvement of enamel morphology is remarkably observed for exposed samples supplemented with F0.05% and F2% as illuminated in Fig. 11b and c. The combination of exposure to PMF and supplement of ChNPs and fluoride indicated the enamel surface of teeth samples to become more homogeneous and densely packed with minerals, as shown in Fig. 11d and e.

### EDX spectroscopy for teeth samples

EDX analysis revealed significant changes in elemental content, specifically the calcium and phosphate concentrations. The former findings are confirmed by EDX results as tabulated in Table 1. The elemental composition of sound teeth showed normal Ca and P concentration ranges of (30.923 ± 0.633) and (16.707 ± 0.315) respectively, while demineralized samples showed a remarkable decrease of its concentrations to be (20.025 ± 0.375) and (11.990 ± 0.280) respectively. The treatment enhances the deposition of minerals and stimulates hydroxyapatite formation, as noticed in the restoration of Ca and P concentration ranges to normal ones. From the given results it is noticed that the highest Ca/P ratio is obtained for teeth samples supplemented by ChNPs-F-0.05% and exposed to PMF than other teeth samples.

**Table 1:** List of Ca and P concentrations percentages and Ca/P ratios for teeth samples with its standard deviation (**± SD)** and ***P***-values.

	Ca%	± SD	*P*- value	*P*%	±SD	*P*- value	Ca/*P*	± SD	*P*- value
Sound	30.923	0.633	-----	16.707	0.315	-----	1.851	0.011	-----
Demineralized	20.025	0.375	-----	11.990	0.280	-----	1.670	0.011	-----
ChNPs	33.480	0.220	0.0771^NS^	17.785	0.055	0.0491*	1.883	0.026	0.0999^NS^
NaF-0.05%	33.445	0.865	0.0554^NS^	17.085	0.355	0.0053**	1.958	0.014	0.0465*
NaF-2.0%	32.460	0.030	0.0983^NS^	16.830	0.150	0.0991^NS^	1.929	0.022	0.0818^NS^
ChNPs-NaF − 0.05%	34.475	1.165	0.0412*	17.015	0.375	0.0331*	2.026	0.034	0.0071**
ChNPs-NaF − 2%	35.715	0.255	0.0471*	18.700	0.100	0.0665^NS^	1.910	0.005	0.0442*
ChNPs-PMF	41.035	5.695	0.0075**	17.850	2.250	0.0910^NS^	2.299	0.042	0.0452*
NaF-0.05%-PMF	38.685	1.665	0.1004^NS^	15.760	0.210	0.0422*	2.455	0.103	0.0061**
NaF-2.0%-PMF	35.430	3.544	0.0711^NS^	15.790	1.470	0.1121^NS^	2.244	0.027	0.0477*
ChNPs-F-0.05%-PMF	43.277	1.879	0.0096**	17.092	0.524	0.0089**	2.532	0.091	0.0092**
ChNPs-NaF − 2%-PMF	46.282	0.556	0.0231*	20.439	1.762	0.0382*	2.264	0.215	0.0411*

^NS^Not Significance > 0.05.

* Significance at level of < 0.05.

** Significance at level of < 0.01.

### Hardness evaluation for teeth samples

In order to differentiate between the surface hardness of various samples, Vickers hardness numbers (VHN) are maintained for all enamel teeth samples, and their values are tabulated in Table 2. The VHN for sound teeth found to be (197.670 ± 11.590) whereas the demineralized teeth VHN is reduced by 45% to be (108.670 ± 7.020). The obtained VHN for all treated teeth indicated remarkable enhancement in the hardness as compared to demineralized specimens. However, unexposed teeth specimens didn’t show similar VHN as sound ones, while the exposed ones were almost similar and/or higher. Significantly, the highest VHN is (275.670 ± 9.020) for samples treated by ChNPs-F-0.05% and exposed to PMF and found to be 39.46% higher than sound tooth. The maximum hardness improvement followed by teeth samples treated by ChNPs-F-2% and exposed to PMF is (246.000 ± 10.600) and found to be 24.45% higher than sound tooth.

**Table 2:** List of VHN values for teeth samples with its standard deviation (**± SD)** and ***P***-values.

	VHN	±SD	*P*- value
Sound	197.670	11.590	-----
Demineralized	108.670	7.020	-----
ChNPs	144.670	14.930	0.0596^NS^
NaF-0.05%	153.470	2.650	0.0066**
NaF-2.0%	152.330	5.270	0.0048*
ChNPs-NaF − 0.05%	144.670	3.670	0.0553^NS^
ChNPs-NaF − 2%	150.670	6.430	0.0074**
ChNPs-PMF	202.600	16.640	0.0599^NS^
NaF-0.05%-PMF	196.330	2.520	0.0035*
NaF-2.0%-PMF	188.670	9.500	0.0801^NS^
ChNPs-F-0.05%-PMF	275.670	9.020	0.0089**
ChNPs-NaF − 2%-PMF	246.000	10.600	0.0019*

^NS^Not Significance > 0.05.

* Significance at level of < 0.05.

** Significance at level of < 0.01.

## Discussion

This research aimed to explore the synergistic remineralization effect for a total of 72 premolar teeth samples by harnessing ChNPs, NaF, and exposure to PMFs. The ChNPs were synthesized by the ionic interaction method, whereas the interaction between TPP and NH3 + of chitosan molecules has resulted in the formation of ChNPs. The SEM micrographs confirmed the well-distributed ChNPs of circular shape in the nano-size diameter and the interspersing of fluoride atoms over the scanned sections. The obtained peaks from XRD analysis of ChNPs substantiated the high degree of crystallinity^25^. The intensity of diffraction peaks was increased as a consequence of transforming amorphous chitosan into crystallized form after reaction with TPP^26^. No other crystalline phases detectable by XRD were found indicating the purity of NaF^27^. On the other hand, the incorporation of NaF into ChNPs tends to exhibit broader and less intense peaks in XRD, suggesting reduced crystallinity and possible amorphous characteristics^28^. The FTIR spectroscopy further provided ChNPs spectrum aided in confirming purity and identifying contaminants, as sharp and well-defined peaks correspond to a high-purity sample^16,29^. FTIR spectroscopy also was employed to analyze the vibrational characteristics of the NaF as shown in the infrared absorption that mainly arises from the stretching and bending of Na-F bonds. Specifically, the addition of NaF reflected a reduction in the peak intensities as illuminated in the sodium fluoride’s spectrum shown in Fig. 7c and d^24^.

Multiple analytical techniques, including XRD, SEM, EDX, and Vickers hardness testing, were employed to assess the morphological, crystallographic, elemental, and mechanical changes in enamel structure across the treated groups. The obtained XRD patterns indicated broad and diffuse diffraction peaks as a result of enamel demineralization in a way that it disrupted the crystalline organization of hydroxyapatite. Furthermore, the crystallographic insights obtained from XRD of treated enamel samples showed minor improvements after treatment with ChNPs alone, but when combined with PMF exposure, the XRD peaks shifted closer to those of sound enamel. The characteristic diffraction peaks showed that the samples’ apatite crystal lattice was partially recovered, completing the process of remineralization as a result of the exposure to PMF compared to those unexposed ones^30,31^. Particularly, a distinct change was evident in the treated teeth sample shown in Fig. 8j, highlighting the effect of mixing ChNPs, NaF 0.05% and exposure to PMF. It showed the diffraction peaks are more sharpened, split, and clear, which matched well with that of the sound tooth structure, suggesting that more apatite crystallinity had formed on the samples’ enamel surface^32^.

Further, the morphological regenerations of demineralized enamels shown in SEM images substantiated the mineral deposition and surface repair inferred from XRD results. The SEM photograph of sound enamel shown in Fig. 9a illuminates dense lines as enamel rods that run from dentine in a slightly curved pattern to the enamel surface^33^. The demineralized teeth sample etched by EDTA showed loss of enamel rod peripheries with intact rod cores that led to wide spaces of its interprismatic distance as a result of the corrosion patterns. In addition, the effect of EDTA on enamel is that it leaches out the positively charged ions and provides a porous, sponge- like-appearing, roughened surface that is negatively charged, as presented in the microporosities due to loss of rod cores and relatively intact periphery^34–36^. The ChNPs have the ability to act as bioadhesive scaffolds, aiding in the controlled release and facilitating the deposition of minerals for further repair of enamel microstructure, as shown in Fig. 10a, d and e^37,38^. The impact of fluoride supplements is clearly observed on the presence of aggregated nanocrystals and smoother surface topography in treated groups indicates an enhanced remineralization process, as shown in Fig. 10b and c. It promotes the deposition of calcium fluoride on the enamel surface which in turn decreases the surface roughness^39^. The combined treatment with ChNPs and fluoride led to the formation of a more compact and uniform mineralized layer making it less prone to caries development as reported elsewhere^40^. The exposure to PMF influences the teeth structures including dental remineralization by decreasing the pH and causing more protonation that leads to ChNPs adhering to the demineralized enamel surface^41^. It promotes deeper penetration and better adherence of positively charged ChNPs to the negatively charged enamel surface, especially under acidic conditions created by demineralization^42^. Synergistically, the PMF has increased ionic uptake of fluoride into the tooth surface and hence improved crystallization processes^43^. The combination of exposure to PMF and supplementation of ChNPs and fluoride indicated the enamel surface of teeth samples to become more homogeneous and densely packed with minerals, as shown in Fig. 11d and e^44^. It shows robust remineralization due to less porous surfaces and pronounced crystalline growth^45,46^. In addition, the pattern of ordered crystals became more oriented to form an organized structure in a way that it simulates the sound tooth structure. It formed new mineral layers, likely fluorapatite, which are more resistant to acid attacks and contribute to the overall hardness and durability of the enamel^47^.

The EDX spectral lines are monitored for calcium and phosphate concentrations as an indicator for the mechanical characteristics of teeth samples. Whereas the amount of calcium and phosphate elements is calculated as mass percentages relative to the total mass of tooth constituents. A comprehensive understanding of the relationship between calcium and phosphate concentrations in terms of the Ca/P ratio helps to assess the mineralization status and mechanical structure of the tooth. An optimal Ca/P ratio is associated with better mineralization of teeth and bones, while imbalances can lead to issues such as dental caries or periodontal disease. Therefore, the Ca/P ratios are calculated for all tooth samples and average values are obtained as listed in Table 1. Studies suggest that an increased Ca/P ratio can enhance the remineralization process, potentially leading to harder and more resilient enamel that can result in the formation of more densely packed and larger hydroxyapatite crystals. A Ca/P ratio approaching or exceeding 1.67 is indicative of sound mineralized enamel and enhanced structural stability^48,49^. Such improved crystallinity and density can enhance the mechanical properties of enamel, including hardness; hence, the higher Ca/P ratios may promote faster nucleation and growth of hydroxyapatite crystals. Enhanced mineral deposition from solutions with higher Ca/P ratios can fill in demineralized zones more effectively, leading to stronger enamel^49^. Significantly, the results indicated changes in calcium and phosphate content, with Ca/P ratios increasing in treatment groups, particularly in the ChNPs + NaF 0.05% + PMF group. Even low levels of fluoride can maintain a steady supply of fluoride ions to the enamel surface, supporting ongoing remineralization and inhibiting demineralization^50^. Specifically, exposure to PMF causes the movement of minerals within the enamel, promoting faster and more effective remineralization, although the exact mechanisms remain under investigation^51^. Fluoride consumption has been shown to have both positive effects on lowering the prevalence of dental cavities and negative effects on tooth enamel and skeletal fluorosis after extended high exposure^52^. These findings are in line with results reported by Richard J.M. Lynch et al. (2014), who showed that the decrease in enamel demineralization and fluoride uptake to a certain limit had a log-linear relationship, above which there was no significant reduction in demineralization^53^. The argument was most likely due to the formation of CaF_2−_like deposits formed on enamel surfaces. Which are thought to act as a protective barrier on the surface as well as serve as a fluoride reservoir. The presence of CaF₂ on the enamel surface may limit the immediate absorption of additional fluoride into the deeper enamel layers. This is because the surface is already saturated with fluoride ions, which reduces the gradient necessary for further fluoride uptake. The results are in resemblance to FA Damato et al., (1990), who investigated the effect of fluoride concentrations on enamel demineralization and remineralization. Remineralization was significantly higher in the lower fluoride compared to the higher concentrations. However, higher fluoride concentrations did not produce any further significant increase in enamel remineralization^54^. The treatment with the ChNPs solution facilitated the deposition of these minerals, helping to rebuild the enamel structure and improving its resistance to further demineralization, as reported elsewhere^55^. The ability to produce fluorapatite crystals and form fluoride-containing (F-C) alkaline compounds around the surface of the enamel that increased enamel resistance maintains successful fluoride incorporation and stops demineralization^56–59^. The harnessing of mixing ChNPs solution with fluoride is the result of the presence of repeating units of β-(1→4)-linked D-glucosamine and N-Acetyl-D-glucosamine in chitosan that includes multiple amino (-NH_2_) and hydroxyl (OH) groups that lead to interaction with various ions and molecules such as fluoride ions (F-). In turn, these interactions enhance the ability of chitosan to adhere to the enamel surface and promote the incorporation of fluoride into the enamel matrix^59,60^. Electrostatically, the adherence of ChNPs to the enamel surface due to their positive charge leads to their being attracted to the negatively charged enamel surface. That turns to create a localized reservoir of fluoride ions on the enamel surface, enhancing the uptake of fluoride into the enamel^61–63^.

One of the most popular methods for identifying changes to the enamel surface in the early phases of caries development is the microhardness test. The microhardness evaluation of dental hard tissues can reveal indirect evidence of mineral gain or loss^64,65^. The surface hardness of tooth enamel could be measured to further investigate dental resistance to abrasion, scratches, and indentation. Additionally, it shows its resistance to permanent curvature and deformation when force is applied^65^. Vickers hardness numbers (VHN) are maintained for all enamel teeth samples as tabulated in Table 2. A marked difference is observed between the treated samples by EDTA and the sound ones, indicating substantial effects from EDTA binding to calcified components of tooth structure through chelating action. Hence, it leads to softening and demineralization of tooth structure^65^. A distinct positive correlation is evident in the treated samples, highlighting the relationship between tooth hardness and its calcium and phosphorus content. In a manner that, as much as the content is high, it gives more strength and hardness. In resemblance to Pancu et al., (2019), who reported that the high remineralization of demineralized tooth structure increases its hardness^66^. There is a clear contrast in the VHN results to the former findings that pronounced improvement is obtained for teeth samples supplemented by ChNPs-NaF-0.05% and exposed to PMF than other teeth samples. The treatment ChNPs-NaF 0.05% and PMF exposure resulted in the ultimate integration of fluoride ions into the enamel in the sample’s remineralization process without leading to excessive hardness or brittleness. The higher concentration of fluoride might lead to excessive fluorapatite formation, which results in a more brittle structure^67,68^. A resulting increasing resistance to indentation and deformation is highlighting the synergistic effect of the exposure to PMF in combination with the remineralizing agents, especially ChNPs-NaF0.05%. Extensively, the resulting synergism arose from the ability of PMF to act as a co-stressor that enhances the penetration of ChNPs into the enamel matrix by altering the tooth surface and improving the absorption of fluoride and other minerals that promote the natural remineralization processes in enamel^11,12,63^. The protocol of exposure to PMF for 10 min 3 times daily for two weeks is nearly simulating the procedure of teeth bleaching, which is affordable for human teeth and referred to as Zoom bleaching^69^. It is an in-office teeth whitening procedure that uses a hydrogen peroxide gel activated by a special light or laser. The application of external exposure accelerates the bleaching by enhancing the breakdown of the peroxide gel. Exposure time of the total procedure usually lasts about 45 min, divided into three to four 15-minute sessions. Light used in Zoom bleaching is typically an LED or a UV light. Moreover, the short-term exposure to PMF was non-ionized radiation of no hazardous effect with very weak magnetic fields aimed to be directed on a localized area just to enhance the mineral uptake^11,12,69^. PMF also can induce an electrophoretic effect, where the electric fields generated move the charged particles (fluoride ions) toward the enamel surface^69^. The enhanced mobility of these particles improves their deposition and incorporation into the enamel. At the same time, it stimulates ionic transport through enamel micro-pores^70^. It helps ChNPs and fluoride ions to move more effectively into the subsurface areas of the enamel. Hence, it may speed up mineral deposition by promoting the crystallization process.

### Limitations and future work

Despite the promising findings, we recognize some limitations and future directions. The present research is performed in an in vitro environment, and future in vivo studies are needed to confirm the results in real-world clinical settings. The current study focused on particular synergistic conditions, and further exploration of varying different PMF exposure parameters involving various concentrations of both ChNPs and fluoride should be performed. Technical challenges, including device accessibility, safety, and patient usability, need to be addressed. Further research is needed to assess the practicality and effectiveness of using PMF devices in domestic settings. Commercially, self- remineralization by the patient to use PMF devices that simulate the (home bleaching) method is recommended to be examined in the future.

## Conclusion

The application of a PMF strategy combined with remineralizing agents significantly enhances dental enamel remineralization. Synergistically, PMF as a co-stressor enhances ChNPs penetration into the enamel while concurrently optimizing NaF uptake. Specifically, the effect of ChNPs and NaF at a low concentration (0.05%) with PMF exposure proved more effective than a higher NaF concentration. This non-invasive technique shows potential for preventing early dental lesions.

## Data Availability

The data is provided within the manuscript.
